# Hippo pathway activation mediates chemotherapy-induced anti-cancer effect and cardiomyopathy through causing mitochondrial damage and dysfunction

**DOI:** 10.7150/thno.79227

**Published:** 2023-01-01

**Authors:** Gang She, Jin-Chan Du, Wei Wu, Tian-Tian Pu, Yu Zhang, Ru-Yue Bai, Yi Zhang, Zheng-Da Pang, Hui-Fang Wang, Yu-Jie Ren, Junichi Sadoshima, Xiu-Ling Deng, Xiao-Jun Du

**Affiliations:** 1 Department of Physiology and Pathophysiology, School of Basic Medical Sciences, Xi'an Jiaotong University Health Science Center, 76 West Yanta Road, Xi'an, 710061, Shaanxi, China.; 2 Department of Pathology, Xi'an People's Hospital (Xi'an Fourth Hospital), Affiliated Guangren Hospital, Xi'an Jiaotong University Health Science Center, 21 Jiefang Road, Xi'an, 710005, Shaanxi, China.; 3 Cardiovascular Research Centre, School of Basic Medical Sciences, Xi'an Jiaotong University Health Science Center, 76 West Yanta Road, Xi'an, 710061, Shaanxi, China.; 4 Baker Heart and Diabetes Institute, 75 Commercial Road, Melbourne, Victoria 3004, Australia.; 5 Rutgers New Jersey Medical School, Department of Cell Biology and Molecular Medicine, New Jersey, United States of America.

**Keywords:** Hippo pathway, mitochondrial damage, anti-cancer chemotherapy, doxorubicin, verteporfin, cardiotoxicity, cardiomyopathy

## Abstract

**Rationale:** Chemotherapy is a common clinical strategy for cancer treatment. However, the accompanied cardiomyopathy renders cancer patients under risk of another life-threatening condition. Whereas Hippo pathway is known to play key roles in both cancerogenesis and heart disease, it remains unclear whether Hippo pathway activation mediates chemotherapy-induced cardiomyopathy.

**Methods and Results:** In human breast cancer cells, doxorubicin (DOX) significantly induced upregulation of Hippo kinase Mst1, inhibitory phosphorylation of YAP, mitochondrial damage, reduced cell viability and increased apoptosis. Hippo pathway inactivation by Mst1-siRNA transfection effectively improved cell survival and mitigated mitochondrial damage and cell apoptosis. Another anti-cancer drug YAP inhibitor verteporfin also induced lower cancer cell viability, apoptosis and mitochondrial injury. Chronic treatment with DOX *in vivo* (4 mg/kg/week for 6 weeks) caused mitochondrial damage and dysfunction, oxidative stress and cardiac fibrosis, while acute DOX treatment (16 mg/kg single bolus) also induced myocardial oxidative stress and mitochondrial abnormalities. Chronic treatment with verteporfin (2 months) resulted in cardiomyopathy phenotypes comparable to that by chronic DOX regimen. In transgenic mice with cardiac overexpression of kinase-dead mutant Mst1 gene, these adverse cardiac effects of DOX were significantly attenuated relative to wild-type littermates.

**Conclusions:** Anti-cancer action of both DOX and verteporfin is associated with Hippo pathway activation. Such action on cardiac Hippo pathway mediates mitochondrial damage and cardiomyopathy.

## Introduction

Currently cancer is the second leading cause of human death worldwide. In addition to surgery and radiation, chemotherapy is an effective strategy for anti-cancer therapy 1. Many classes of anti-cancer drugs are widely used while new agents are continuously under development. Despite the therapeutic benefits of chemotherapy, cardiotoxicity in cancer patients has been increasingly taken seriously 2. Cardiotoxicity outcomes are responsible for 7%-27% of cardiovascular mortality in cancer patients 3. Alongside of its common use, doxorubicin (DOX) is the most frequently reported drug in chemotherapy-induced cardiotoxicity 4. Numerous experimental studies in DOX-treated rodents have reported severe left ventricular (LV) dysfunction and cardiomyopathy examined by echocardiography and cine magnetic resonance 5, 6. In addition, cancer patients receiving DOX treatment often exhibited similar conditions from asymptomatic LV dysfunction to cardiomyopathy and progressive heart failure (HF) 7, 8. It is an urgent unmet need to develop preventive and therapeutic strategy for cancer patients to battle serious cardiac complications. However, the molecular mechanism of cardiotoxicity by chemotherapy remains less explored.

The Hippo pathway is a highly conserved signaling pathway, which plays a pivotal role in cell fate, organ size control and tissue homeostasis 9. Hippo pathway is composed of a kinase cascade consisting of mammalian sterile 20-like 1 (Mst1) and large tumor suppressor homolog (Lats), and the downstream transcriptional coregulator yes-associated protein (YAP). Nuclear YAP interacts with its transcriptional factor TEA-domain family member 1 (TEAD1) and regulate expression of numerous genes 10. Activation of Mst1-Lats cascade induces YAP phosphorylation at Ser^127^ site (Ser^127^-pYAP), limiting nuclear localization of YAP and inactivation of its transcriptional regulation. In cancer cells, Hippo pathway is usually inactivated permitting an enhanced transcriptional activity of YAP 11, which facilitates proliferation, migration and invasion of cancer cells 12. Indeed, in various cancer types, YAP expression and nuclear localization of tissue biopsies are associated with poor prognosis 13, 14, and chemotherapy-induced suppression of cancer growth is associated with inhibition of oncogenic activity of YAP 12, 15. Accordingly, YAP inhibitor verteporfin has recently been developed as a chemotherapy drug that binds to TEAD-binding domain of YAP thereby blocking YAP-TEAD1 interaction with curative effect on a variety of malignancies, albeit its potential cardiotoxicity remains largely unknown 16.

Recent studies have revealed a pivotal role of the Hippo pathway in heart disease 17. In a variety of diseased conditions like HF, myocardial infarction and dilated cardiomyopathy (DCM), Hippo pathway is activated while YAP activity is inhibited 10, 18. Using a mouse model of DCM owing to enhanced cardiac Hippo pathway via cardiomyocyte-restricted Mst1 overexpression (Mst1-TG) 19, 20, we recently demonstrated mitochondrial dysfunction and metabolic disturbance as a causative factor in the development of DCM and HF 21. Similar mitochondrial dysfunction and DCM phenotype have also been reported in mice with cardiomyocyte YAP or TEAD1 gene deletion 22-24. These findings implicate a key role of Hippo pathway in governing cardiac mitochondrial homeostasis and function. Whether chemotherapy-induced cardiomyopathy is associated with activation of cardiac Hippo pathway has not been investigated.

Here we studied the hypothesis that anti-cancer drugs activate Hippo pathway in cancer cells and cardiomyocytes with resultant anti-cancer efficacy but cardiotoxicity, and that mitochondrial damage and dysfunction form the underlying mechanism. To test this hypothesis, two anti-cancer drugs, DOX and verteporfin, were tested in human breast cancer cells with or without Mst1-knockdown, and in wild-type and Mst1-inactivated mice 25.

## Methods

### Animals and drug treatment

We used C57BL/6J mice and the dnMst1-TG mice together with non-TG littermates (nTG) in the same C57BL/6J genetic background. dnMst1-TG mice carry a kinase‐dead mutant Mst1 (K59R) transgene driven by cardiomyocyte-specific α-myosin heavy chain promoter, achieving overexpression of a dominant‐negative mutant transgene with inactivation of the endogenous Mst1 activity 25. Genotype was determined by PCR of tail biopsy. Animals were housed in standard conditions, and male mice used at 10-12 weeks of age. Animals were randomly grouped into different experiments. All experimental protocols were reviewed and approved by the Institutional Animal Care and Use Committee of Xi'an Jiaotong University and conformed to the Guide for the Care and Use of Laboratory Animals published by the National Institutes of Health, USA.

To simulate DOX administration in the clinic, DOX was tested in mice with two different regimens, acute treatment (16 mg/kg dissolved in saline, i.p., single injection) and chronic treatment (4 mg/kg/week, i.p. once a week for 6 weeks). C57BL/6J mice were administrated with YAP inhibitor verteporfin (100 mg/kg dissolved in corn oil, i.p. once every other day) for a period of 2 months. Control animals received equal volume of solvent. At the end of the study, mice were anesthetized and blood was collected to obtain plasma, hearts were removed for assay.

### Echocardiography

Echocardiography was performed as described previously 26. Using a Vevo 2100 ultrasound machine (Visualsonics Inc, Toronto, Canada) with a MS550D transducer, 2-dimensional image of the LV was obtained at a level of the papillary muscles, and 2-dimensional guided M-mode traces crossing the anterior and posterior walls were recorded. The following parameters were measured in a blinded fashion on the M-mode tracings: LV wall thickness at diastole and systole, LV internal dimensions (LVID) at diastole and systole (LVIDd, LVIDs), ejection fraction (EF) and fractional shortening (FS). Measurements were taken from 5 cardiac cycles, and the averages used.

### Cell culture and RNA interference

MDA-MB-231 human breast cancer cells were obtained from the ATCC. Cells were cultured in high glucose DMEM medium supplemented with 10% FBS and antibiotics. MDA-MB-231 cells grown to approximately 70% confluency were transfected with control or Mst1-siRNA, procedure was as described previously 26. Specific siRNA molecules targeted to Mst1 were synthesized by Gene Pharma Company (Shanghai, China). The siRNA sequences of human Mst1 were as follows: sense: 5'- CCGGCCAGAUUGUUGCUAUUA-3', antisense: 5'-UAAUAGCAACAAUCUGGCCGG-3'. After 6-hour transfection, cells were treated with or without 1 µM DOX for 48 h. After incubation, cells were harvested for analysis.

### Transmission electron microscopy and quantification

Transmission electron microscopy (EM) was applied to observe the ultrastructures of MDA-MB-231 cells or mouse LV tissues, as described previously 21. In brief, cells or fresh tissue blocks (1 mm^3^) were immediately fixed with 2.5% glutaraldehyde. After fixation, the samples were dehydrated by a graded series of ethanol, then dehydrated by alcohol and eventually transferred to absolute acetone. Following infiltration with absolute acetone and the final Spurr resin mixture, the samples were embedded, ultrathin sectioned and stained. Finally, the samples were observed in the transmission electron microscope (Hitachi Model H-7650). For each sample, images from randomly chosen fields were obtained at magnifications of ×4,000 and ×10,000, respectively (6-10 images at each magnification per sample). Analysis of the size and number of mitochondria, the length of sarcomere or density of lipid droplets in images was performed in Image Pro Plus 6.0 software.

### Western blotting analysis

As described previously 27, Polyvinylidene fluoride membranes were incubated with primary antibody (see Table S1) overnight at 4 °C, and then incubated with horseradish peroxidase-conjugated secondary antibodies (1:10000) for 1 h at room temperature. The bound antibodies were detected with an enhanced chemiluminescence detection system (Bio-Rad, USA) and quantified by densitometry using the ImageJ software.

### Immunofluorescent staining

Immunofluorescent staining of heart sections was performed as previously described 27. In brief, LV sections were incubated overnight at 4 ℃ with anti‐YAP antibody (1:200). After incubation with secondary goat anti‐rabbit Cy3 antibody at room temperature for 1 h, the sections were sequentially incubated with wheat germ agglutinin (WGA) and DAPI (4′6-diamidino-2-phenylindole, dihydrochloride) for 10 minutes, separately. Then, images were acquired with Leica TCS SP8 STED 3X confocal microscopy.

### Immunoprecipitation

Immunoprecipitation experiments were performed as previously described 27. In brief, nuclear protein was extracted from mouse LV tissues using nuclear protein extraction kit (Beyotime, Shanghai, China). Cleared lysates were immunoprecipitated with anti-YAP1 or anti-TEAD1 antibody at 4 °C overnight and then coupled to suspended Protein A/G agrose beads (Santa Cruz, CA, U.S.A.). Beads were washed three times in 400 μL lysis buffer. Immunoprecipitates were boiled in SDS-Laemmli buffer and thereafter subjected to Western blotting analysis with anti-TEAD1 or anti-YAP1 antibody.

### Determination of ROS

Cardiac level of ROS was determined by using two independent methods. As we previously described 21, O_2_^-^ level in myocardium was detected with dihydroethidium (DHE) fluorescent stain of LV frozen sections (5 μm) by incubation of DHE (5 µM for 1 h at 37 °C). Images were digitized (×400 magnification, 6 fields per heart) under a fluorescence microscope (DP72; Olympus, Tokyo, Japan) with excitation/emission at 488/610 nm. In addition, chloromethyl derivative CM-H2DCFDA (DCF) fluorescent staining of isolated adult mouse cardiomyocytes was used to test ROS level in the myocardium. Cardiomyocytes isolated through Langendorff perfusion system were incubated with 5 µM DCF for 30 min at 37 ℃. Images were obtained using a Leica TCS SP8 STED 3X confocal microscope with 40× 1.3 NA oil immersion objective (excitation 488 nm, emission 525 nm) with fixed scanning parameters. An average of 50 cells of each heart were analyzed for the intensity of DCF fluorescence in ImageJ software.

### RNA sequencing and bioinformatics

Transcriptome sequencing of extracted RNA from LV tissues of DOX treated mice was performed by BioTree Shanghai (http://www.biotree.com.cn/). After mRNA enrichment, strand-specific libraries were constructed using the TruSeq RNA sample preparation kit (Illumina, San Diego, CA, USA), and sequencing was carried out using the Illumina Novaseq 6000 instrument yielding 150 bp paired end reads. The raw data were handled by Skewer and data quality was checked by FastQC v0.11.2. Reads were mapped to gene exons using Hisat2 v2.0.5 software for genome annotation, and genes with less than 20 reads/sample were excluded from further analysis. The differentially expressed transcripts (DETs) were computed with DESeq2 package (version 1.20.0) supplied by R software (version 3.4.4). Data were then subjected to functional enrichment analysis by STRING software (version 10.5). Genes and gene sets with a false discovery rate (FDR) adjusted p-value < 0.05 were considered statistically significant. Statistical significance of gene sets was determined by a multivariate ANOVA (MANOVA). FDR adjustment with the Benjamini-Hochberg method was used after edgeR and MANOVA tests.

### Statistical analysis

All data were presented as mean ± SEM. Results were analysed using GraphPad Prism 8 software. Statistical comparisons among groups were performed by one-way analysis of variance (ANOVA) with Bonferroni's post hoc test. Two-way ANOVA for repeated measures followed by the Bonferroni's post hoc test was used to analyze cell viability differences over time among groups. A Student's t test was used to compare differences between only two groups. Nonparametric test was used to analyze differences among groups of EM-derived parameters that were not normal distribution. Statistical comparisons between two groups were performed by Mann-Whitney U test, and Kruskal-Wallis test followed by the Dunn's multiple comparison test for among more than two groups. A value of *P* < 0.05 was considered to be significantly different.

### Materials

Information of antibodies was documented in Table S1. DOX and verteporfin were from MedChem Express (USA). TUNEL stain kit, CCK-8 kit, DHE and DCF reagents were purchased from Beyotime Institute of Biotechnology (Shanghai, China). ATP assay kits was obtained from Jiancheng Bioengineering Institute (Nanjing, China). ELISA kits for plasma cTnI and Gal-3 determination and lactate assay kit were from Elabscience Biotechnology Co., Ltd (Wuhan, China).

## Results

### DOX-induced apoptosis and reduced cancer cell viability were indispensable to Hippo pathway activation

**Figure 1:**
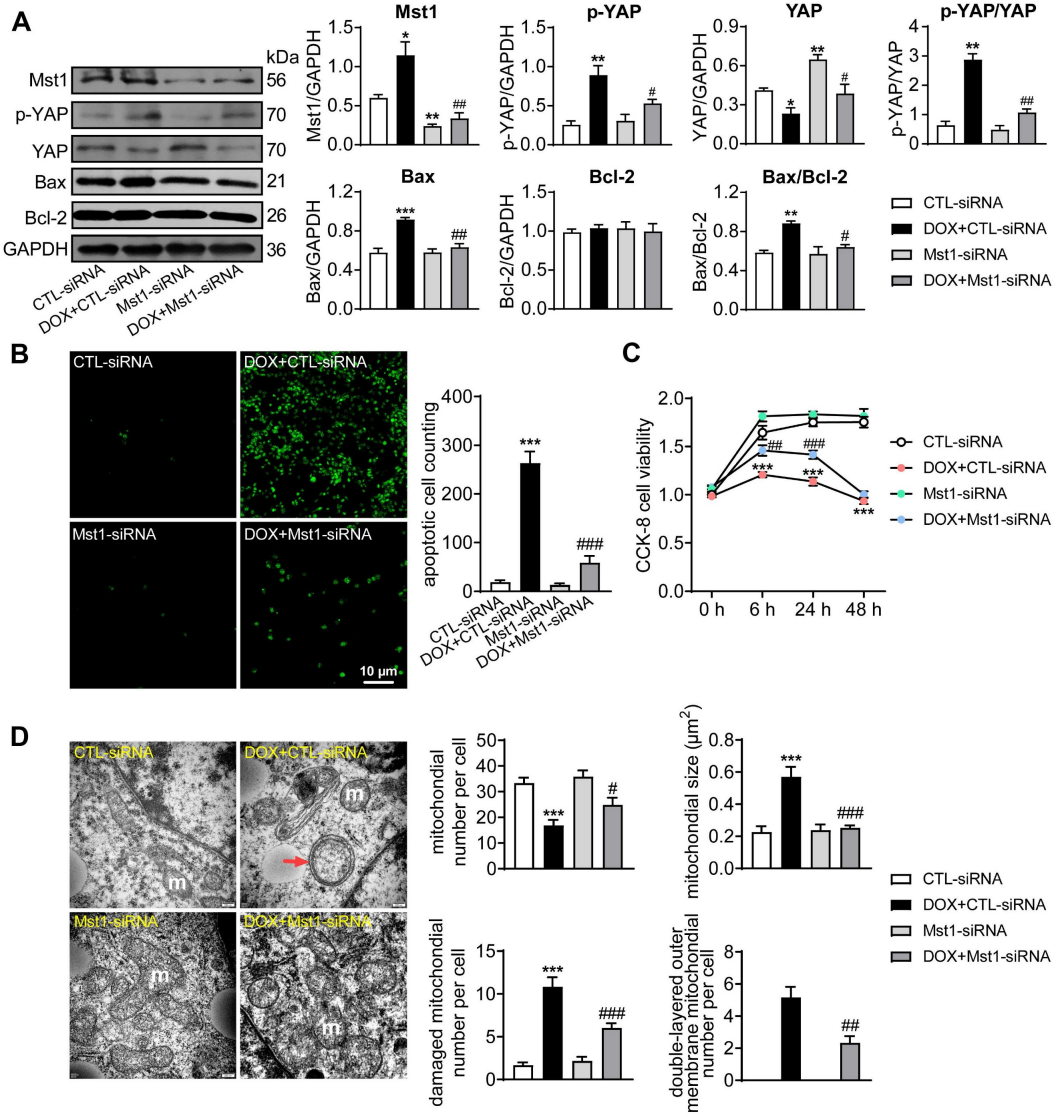
** Cancer cell apoptosis and alterations in cell viability and mitochondrial ultrastructure induced by treatment with doxorubicin (DOX).** MDA-MB-231 human breast cancer cells were transfected with control siRNA (CTL-siRNA) or Mst1-siRNA (50 nM), and then cells were treated with or without DOX (1 μM) for 48 h. **(A)** Representative immunoblotting images and quantitative analysis for protein expression level of Mst1, YAP, Ser^127^-pYAP, Bax and Bcl-2 in cells with siRNA transfection and DOX stimulation, data is expressed as ratio relative to GAPDH (n = 6). **(B)** Images and quantitative analysis of TUNEL staining in MDA-MB-231 cells treated with the interventions as described in A (n = 6). **(C)** Cell viability tested by CCK-8 kit at different time points after siRNA transfection and/or following DOX stimulation (n = 12). **(D)** Electron microscopic (EM) images (magnification at 10,000) showing mitochondrial ultrastructure and quantitative analysis of mitochondrial number and size, damaged (membrane incomplete or cristae dissolved) mitochondrion count and the amount of mitochondria with double-layered mitochondrial outer membrane. Results were counts in per cell; m: mitochondria, red arrow denotes condensed and/or double-layered mitochondrial outer membrane (n = 10 cells per group). Data are presented as Mean ± SEM, ^*^P < 0.05, ^**^P < 0.01, ^***^P < 0.001* vs.* CTL-siRNA; ^#^*P* < 0.05, ^##^*P* < 0.01, ^###^*P* < 0.001 *vs.* DOX+CTL-siRNA.

The anti-cancer effect of DOX was investigated in human breast cancer cells (MDA-MB-231) with the role of Hippo pathway tested by Mst1-knockdown using Mst1-siRNA. DOX (1 µM) stimulation significantly activated Hippo pathway in cancer cells manifested as increased levels of Mst1 protein and Ser^127^-pYAP while total YAP expression decreased. Silencing Mst1 gene through Mst1-siRNA transfection reduced Mst1 protein expression by 65%, which effectively inhibited DOX-induced Hippo pathway activation (Figure 1A). In addition, immunoblotting and TUNEL stain in DOX-treated cancer cells revealed upregulation of mitochondrial pro-apoptotic protein Bax, higher ratio of Bax/Bcl-2, and a 10-fold increase in cell apoptosis (Figure 1A-B). Cell viability tested by CCK-8 reagent also revealed significant decrease after 6 h onwards following DOX stimulation (Figure 1C). By EM imaging, cancer cells treated with DOX showed significant mitochondrial damage evidenced by decreased mitochondrial number, increased mitochondrial membrane density, swelling and cristae partial dissolvement, and condensed or double-layered outer membrane (Figure 1D). These changes by DOX were largely prevented by Mst1 silencing. These data implicated that DOX could induce cancer cell mitochondrial damage, viability decrease and apoptosis in a Hippo pathway dependent manner.

### Verteporfin reduced cancer cell viability and induced apoptosis through inhibiting YAP activity

**Figure 2:**
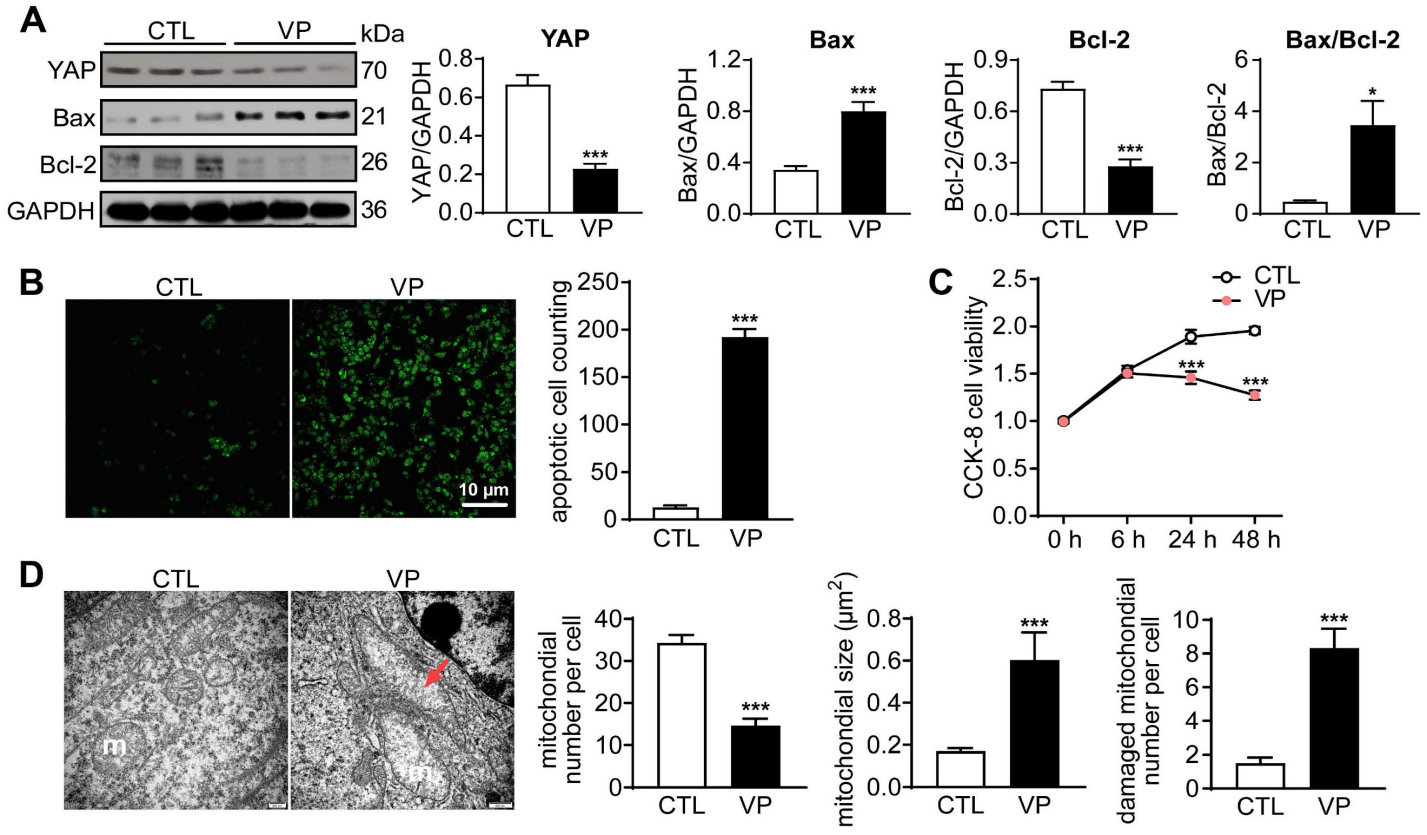
** Cancer cell apoptosis and alterations in cell viability and mitochondrial ultrastructure after stimulation with YAP-inhibitor verteporfin (VP). (A)** Representative immunoblotting images and quantitative analysis for YAP, Bax and Bcl-2 protein expression level in cells with or without VP (1 μM) for 48 h, data is expressed as ratio relative to GAPDH (n = 6). **(B)** Images and quantitative analysis of TUNEL staining in cells treated with or without verteporfin (n = 6). **(C)** Cell viability at different time points after verteporfin stimulation tested by CCK-8 kit (n = 12). **(D)** EM images showing mitochondrial structure alterations by verteporfin treatment and quantitative analysis of mitochondrial number, size and damaged (membrane incomplete or cristae dissolved) mitochondrion count, magnification at 10,000; m: mitochondria, red arrow denotes swollen and cristae-dissolved mitochondria (n = 10 cells per group). Data are presented as Mean ± SEM, ^*^P < 0.05, ^***^P < 0.001* vs.* control (CTL).

Another anti-cancer drug YAP inhibitor verteporfin was similarly tested in human breast cancer cells. Incubation with verteporfin (1 µM) significantly decreased expression of YAP and Bcl-2, but increased Bax expression, changes accompanied by increased cell apoptosis (about 14-fold increase) and reduced viability (about 50%, Figure 2A-C). In verteporfin-treated cells, EM images also showed pronounced mitochondrial injury, manifested by mitochondrial number decrease, membrane incomplete, swelling and cristae dissolution (Figure 2D). These data demonstrated that YAP inhibition by verteporfin could induce cancer cell mitochondrial damage, viability decrease and apoptosis.

### DOX treatment downregulated expression of cardiac mitochondrial gene sets

**Figure 3:**
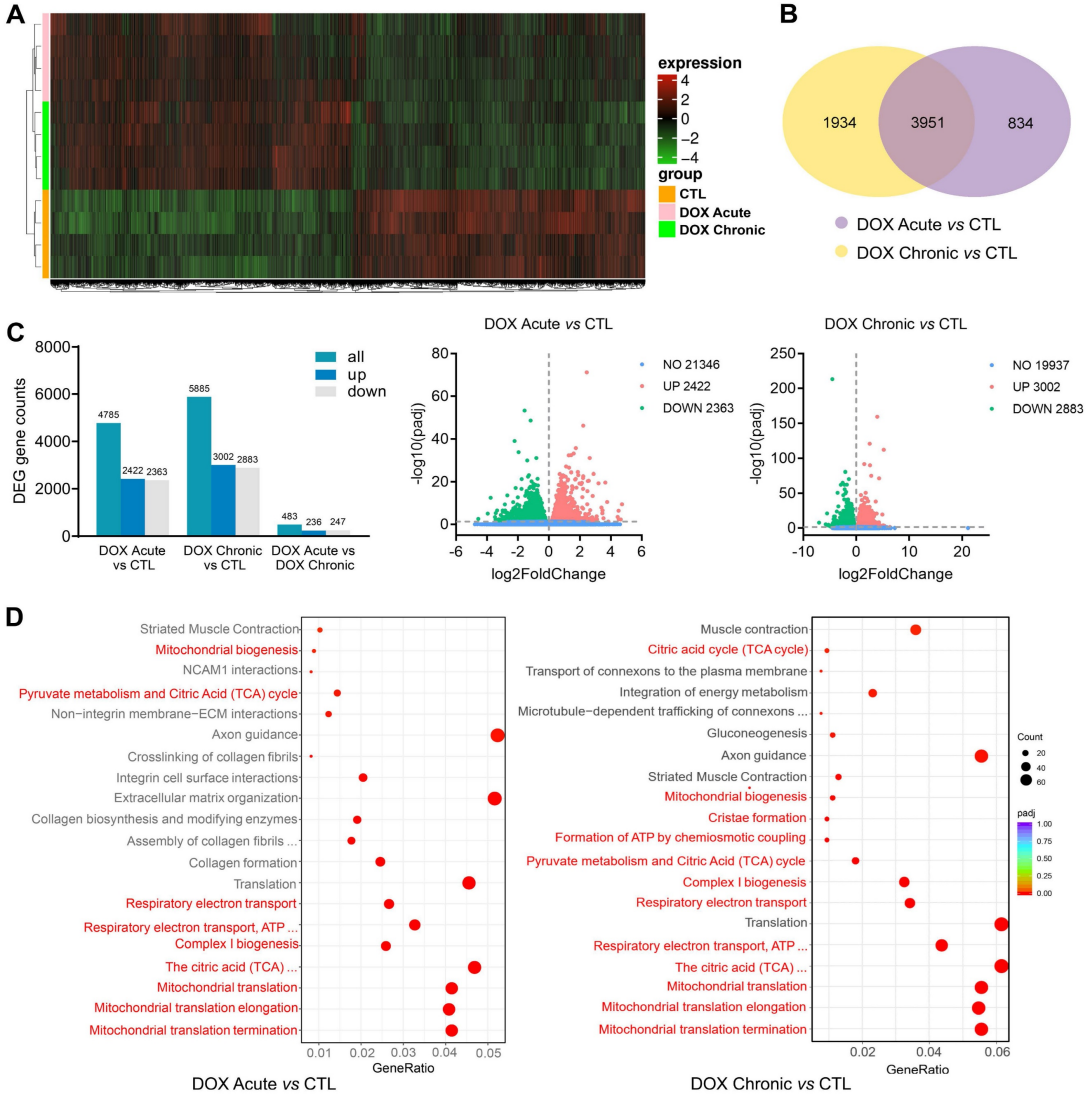
** Alterations in transcriptome and Reactome enrichment analysis by RNA-sequencing in DOX treated C57BL/6 mice with different regimens.** C57BL/6 mice were administrated with or without different doses of DOX (acute: 16 mg/kg, i.p., single injection; chronic: 4 mg/kg/wk, i.p., once a week for 6 weeks). RNA-seq data were collected from LV tissues of mice (n = 4 in each group). **(A)** Heatmap showing changes in gene clusters among groups.** (B)** Venn diagram of number of differentially expressed genes (DEGs) relative to control group. **(C)** Counts of DEGs and volcano plots of total RNA-sequencing showing DEGs among groups. **(D)** Bubble plots of Reactome enrichment analysis for the most significantly down-regulated gene sets by DOX treatment. Red color denotes mitochondrion related gene sets.

Alterations in cardiac transcriptome by different regimens of DOX treatment were explored by using RNA-sequencing and bioinformatics. RNA-seq detected approximately 26,000 genes, in which about 18% were differentially expressed genes (DEGs) induced by acute DOX-therapy with equal number of genes up- or down-regulated (2,422 *vs* 2,363). In chronic DOX treated hearts, about 23% DEGs were identified (up: 3,002, down: 2,883) (Figure 3A-C). There was certain degree of overlapping in DEGs between acute and chronic DOX groups with 3,951 DEGs simultaneously detected in both DOX groups (Figure 3A). However, 1,934 DEGs presented only in chronic DOX group and 834 DEGs only occurred in acute DOX group (Figure 3B). Reactome pathway enrichment analysis showed that of 2,774 detected gene sets in acute DOX group, 502 (18%) were differentially expressed. In chronic DOX group, 649 out of a total of 2,234 gene sets (29%) were differentially expressed. Notably, mitochondria associated gene sets were among the top-20 most significantly down-regulated gene sets (Figure 3D), including citric acid cycle, mitochondrial biogenesis, ATP synthesis, mitochondrial complex, respiratory electron transport and mitochondrial translation.

### DOX therapy activated cardiac Hippo pathway

**Figure 4:**
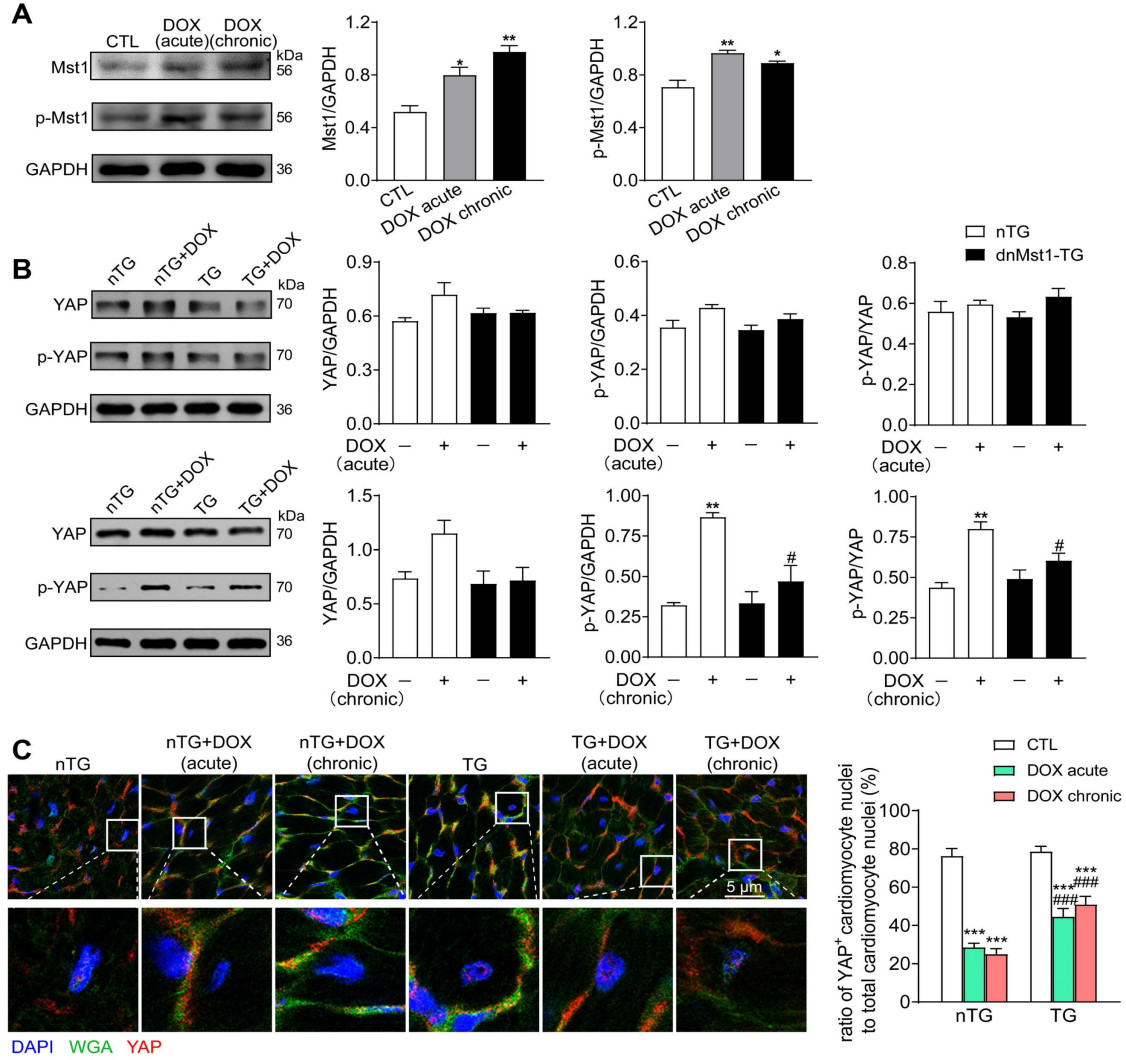
** Altered expression of Hippo pathway components induced by DOX treatment in nTG and dnMst1-TG mice.** nTG (non-transgenic) and dnMst1-TG (dominant negative Mst1 transgenic) mice were treated with or without different doses of DOX (acute: 16 mg/kg, i.p., single injection; chronic: 4 mg/kg/wk, i.p., once a week for 6 weeks, 24 mg/kg in total). **(A)** Representative immunoblotting images and quantitative analysis of Mst1 and Thr^183^-pMst1 level in hearts from nTG mice subjected to different DOX regimens. n = 6, ^*^P < 0.05, ^**^P < 0.01* vs.* CTL. **(B)** Representative immunoblotting images and quantitative analysis of YAP and Ser^127^-pYAP level in nTG and dnMst1-TG mouse heart with or without DOX treatment. n = 6, ^**^P < 0.01* vs.* nTG; ^#^*P* < 0.05 *vs.* nTG+DOX.** (C)** Immunofluorescent stain of mouse left ventricles showing the location of YAP (red) in cardiomyocyte nuclei, cell nucleus and membrane were stained with DAPI (4′6-diamidino-2-phenylindole, dihydrochloride) and WGA (wheat germ agglutinin), separately (×1200). High magnification YAP positive cardiomyocyte nuclei in different groups were presented and the number was counted and presented as the ratio of YAP^+^ cardiomyocyte nuclei to total cardiomyocyte nuclei. n = 3 samples per group and 3 images got from each sample were used for statistics, ^***^P < 0.001* vs.* respective control (CTL);^ ###^*P* < 0.001 *vs.* nTG counterpart. Data are presented as Mean ± SEM.

To address the role of Hippo pathway in DOX-mediated cardiomyopathy, nTG and dnMst1-TG mice were subjected to acute or chronic DOX treatment, and parameters of cardiotoxicity and changes in Hippo pathway activity were determined. In nTG mice, both acute and chronic DOX regimens increased expression level of Mst1 and Thr^183^-pMst1 (Figure 4A). Expression of both total YAP and Ser^127^-pYAP was tended to be higher by acute DOX treatment, but significantly increased by chronic DOX treatment (Figure 4B). In dnMst1-TG mouse hearts, Hippo pathway activity was assessed by changes in YAP and Ser^127^-pYAP levels. Whereas the effect of Mst1-inactivation (i.e. dnMst1-TG) on YAP activity was not detected in acute DOX group, significant attenuation of Hippo pathway activity was evident following chronic DOX treatment (Figure 4B). Using immunofluorescent stain, we further detected YAP localization in cardiomyocyte nuclei. As shown in Figure 4C, cardiomyocyte nuclear YAP was significantly decreased after acute or chronic DOX treatment, but in TG mouse hearts, acute and chronic DOX treatment induced myocardial nuclear YAP reduction were effectively mitigated. Our results indicated activation of cardiac Hippo pathway with suppressed YAP transcriptional activity by DOX therapy, in particular the chronic regimen.

### Mst1-inactivation mitigated DOX induced cardiac mitochondrial damage and dysfunction

**Figure 5:**
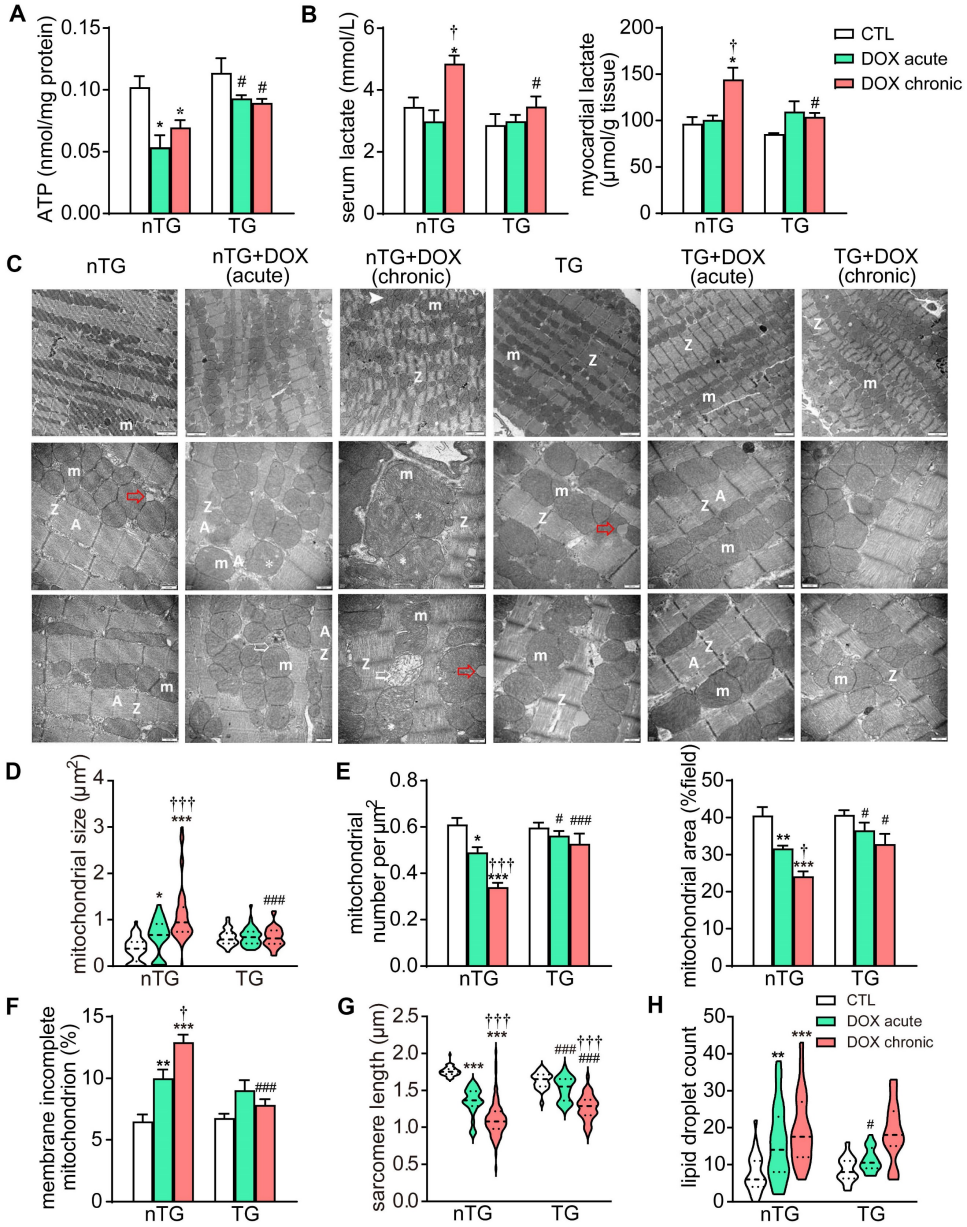
** Changes in myocardial mitochondrial structure and function in nTG and dnMst1-TG mice with different interventions. (A)** ATP level in left ventricular (LV) tissues (n = 6 per group). **(B)** Lactate level in serum and LV tissues (n = 6 per group). **(C)** Electron microscopic (EM) images of LV myocardium. Images were representatives of n = 3 heart per group. From top to bottom: magnification at 4,000, 10,000 and 10,000, respectively. m: mitochondria; A: A band; Z: Z line; ➤: irregularly arranged mitochondia; *: swollen mitochondria or mitochondria with disrupted or dissolved cristae; ➯: damaged mitochondria with incomplete outer-membrane; red arrow denotes lipid droplet. **(D)** Violin plot showing quantitative measures of the size and density of mitochondrion. **(E)** Distribution of mitochondrial size of mouse heart. **(F)** The ratio of mitochondria with incomplete outer-membrane. **(G)** Violin plot showing sarcomere length of mouse heart. **(H)** Violin plot showing lipid droplet count of cardiomyocytes. Data are presented as Mean ± SEM, ^*^P < 0.05, ^**^P < 0.01, ^***^P < 0.001* vs.* respective control (CTL);^ †^*P* < 0.05, ^†††^*P* < 0.001 *vs.* respective DOX Acute; ^#^*P* < 0.05, ^###^*P* < 0.001 *vs.* nTG counterpart.

Based on RNA-seq data revealing downregulation of mitochondrial genes in DOX treated hearts, we further examined changes by DOX treatment in mitochondrial structure and function in mouse hearts. Both DOX regimens significantly decreased ATP level in the myocardium, and the serum and tissue levels of lactate increased by chronic DOX regimen. These metabolic disturbances were significantly blunted in dnMst1-TG mice (Figure 5A-B). EM images from LV tissues (3 hearts per group) were acquired for analysis of ultrastructural changes in mitochondria and sarcomeres. Both DOX regimens resulted in disordered arrangement, swelling, partial cristae dissolution, and loss of outer-membrane integrity of mitochondria (Figure 5C and F). Quantitative analysis showed increase in mitochondrial size by about 50% in acute DOX group and by 3-fold in chronic DOX group (Figure 5D), whilst mitochondrial density measured by number per μm^2^ or area per field, decreased by 15% and 40%, respectively (Figure 5E), indicative of decreased mitochondria number in cardiomyocytes. There was significant shortening of sarcomere length and increase of lipid droplets in both DOX treated groups (Figure 5G and H), likely due to disorder of mitochondrial lipid metabolism and shortage of ATP supply essential for myofiber relaxation in the cardiac cycle. These ultrastructural changes of mitochondria and sarcomeres by acute and chronic DOX regimens were apparently less severe in dnMst1-TG hearts relative to nTG counterparts (Figure 5D-H).

**Figure 6:**
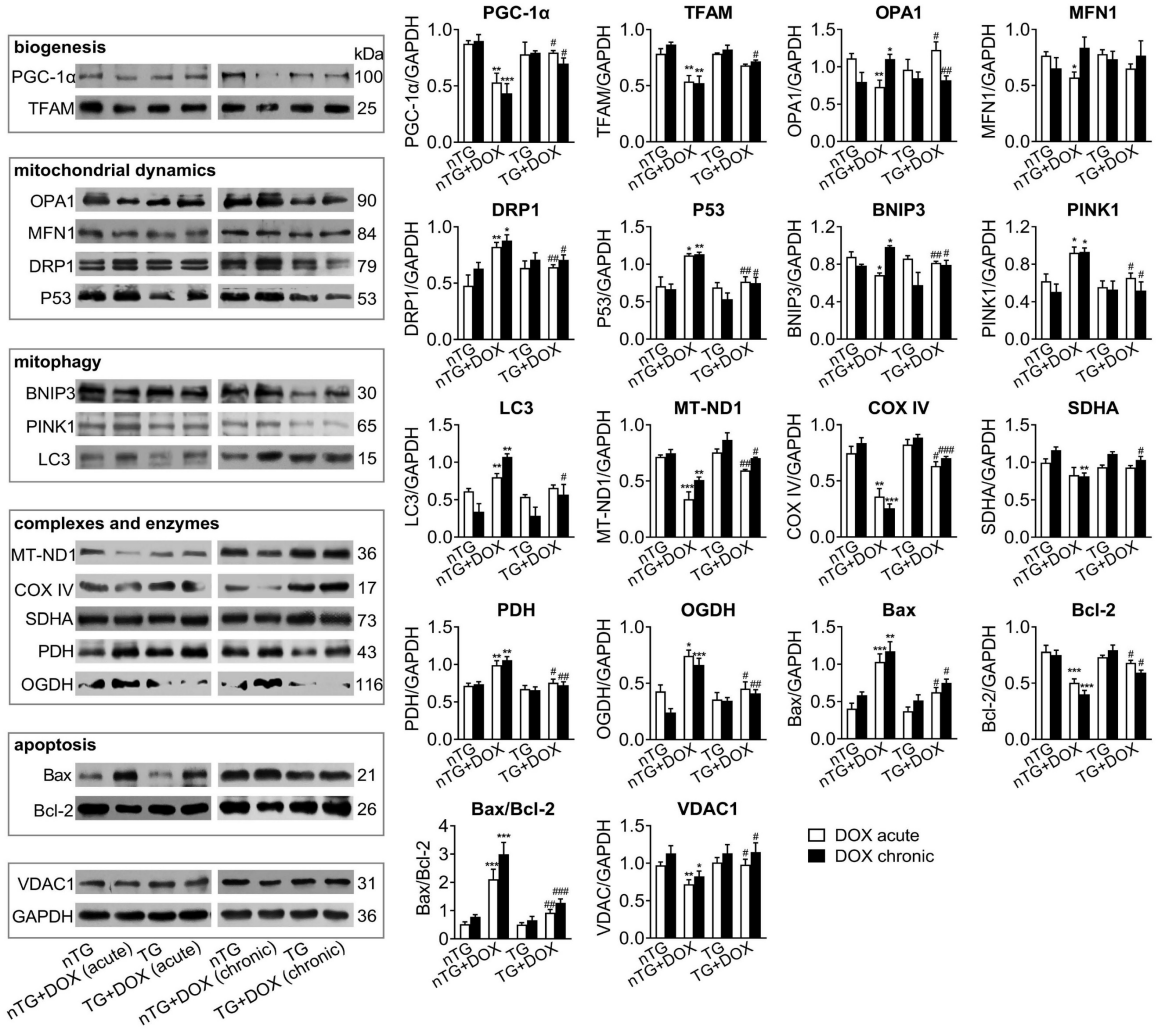
** Expression of mitochondrial marker proteins in nTG and dnMst1-TG hearts with different interventions.** Immunoblotting was performed on protein prepared from mouse LV tissues. Proteins tested were representative markers for mitochondrial biogenesis, fusion, fission, mitophagy, respiratory complexes I-III or TCA cycle enzymes, apoptosis and transporter VDAC1. Protein expression level was quantified and expressed as ratio relative to GAPDH. Data are presented as Mean ± SEM, n = 6, ^*^P < 0.05, ^**^P < 0.01, ^***^P < 0.001 *vs.* nTG; ^#^*P* < 0.05, ^##^*P* < 0.01, ^###^*P* < 0.001 *vs.* nTG+DOX.

Next, we studied in DOX-treated mice alterations in expression of mitochondrial marker proteins for mitochondrial metabolism and dynamics. Immunoblotting results revealed significantly down-regulated PGC-1α and TFAM as mitochondrial biogenesis markers by acute or chronic DOX regimens (Figure 6). Dysregulated mitochondrial dynamics was indicated by increased marker proteins for fission (DRP1 and P53), mitophagy or apoptosis (PINK1, BNIP3, LC3 and Bax/Bcl-2), but reduced proteins for fusion (OPA1 and MFN1). Selected marker proteins for mitochondrial complexes or energy metabolism were markedly altered (Figure 6). Being the most abundant channel protein localized in mitochondria out-membrane, VDAC1 protein level decreased by acute and chronic DOX regimens, a finding in keeping with reduced mitochondrial density by EM imaging. Once again, inactivation of Mst1 (dnMst1-TG) effectively preserved expression of these marker proteins in the setting of acute or chronic DOX treatment (Figure 6).

### Hippo pathway inhibition mitigated DOX-induced cardiac injury, fibrosis and oxidative stress

**Figure 7:**
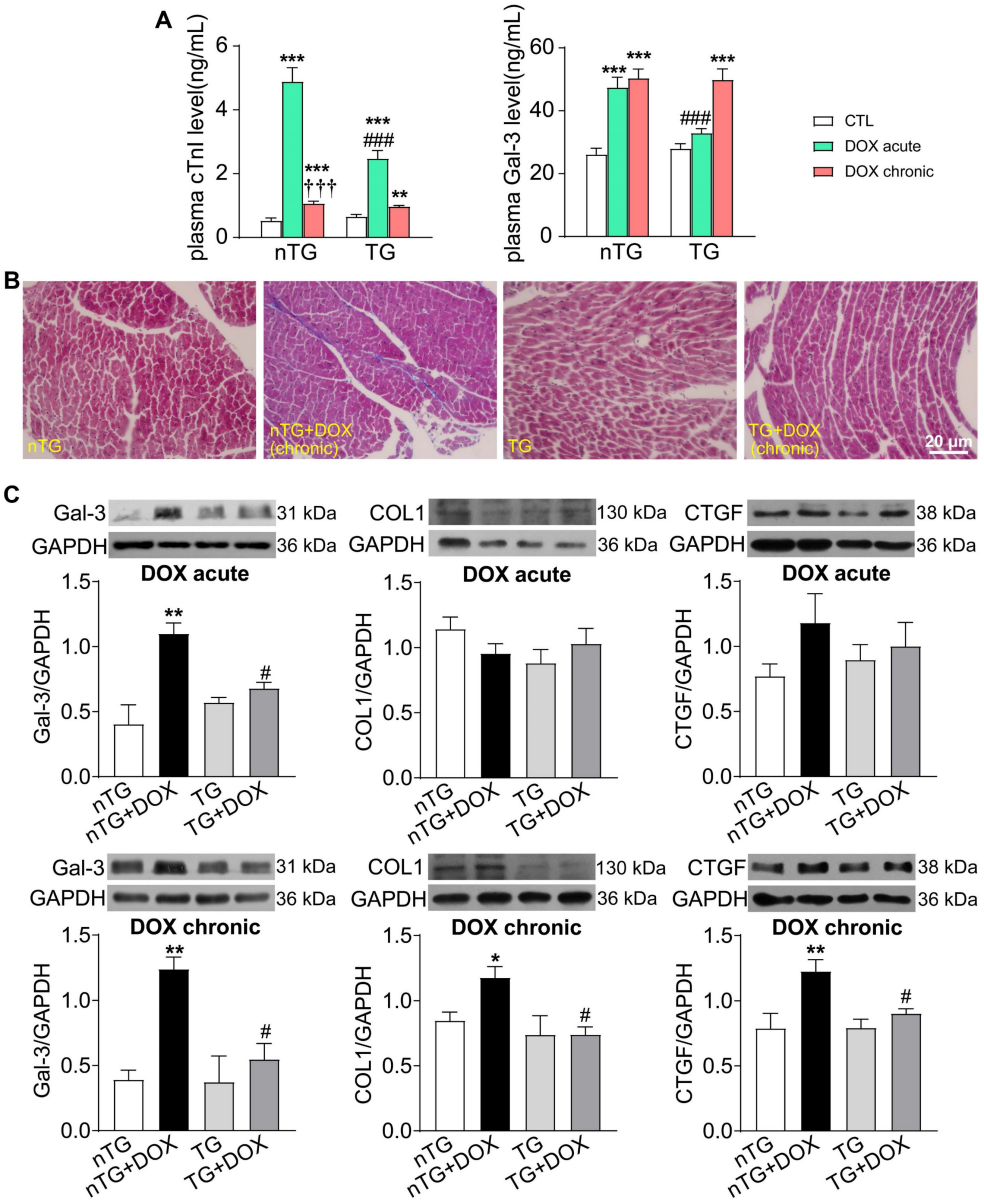
** Changes in markers for cardiac injury and fibrosis induced by DOX treatment with different interventions in nTG and dnMst1-TG mice. (A)** Plasma levels of myocardial injury markers cTnI and Gal-3. n = 6, ^**^P < 0.01, ^***^P < 0.001* vs.* respective control (CTL); ^†††^*P* < 0.001 *vs.* respective DOX Acute; ^###^*P* < 0.001 *vs.* nTG counterpart. **(B)** Masson trichrome staining of LV sections for collagen (blue). **(C)** Representative immunoblotting images and quantitative analysis for protein expression level of Gal-3, collagen I (COL1) and CTGF in LV tissues. n = 6, ^*^P < 0.05, ^**^P < 0.01* vs.* nTG; ^#^*P* < 0.05 *vs.* nTG+DOX. Data are presented as Mean ± SEM.

**Figure 8:**
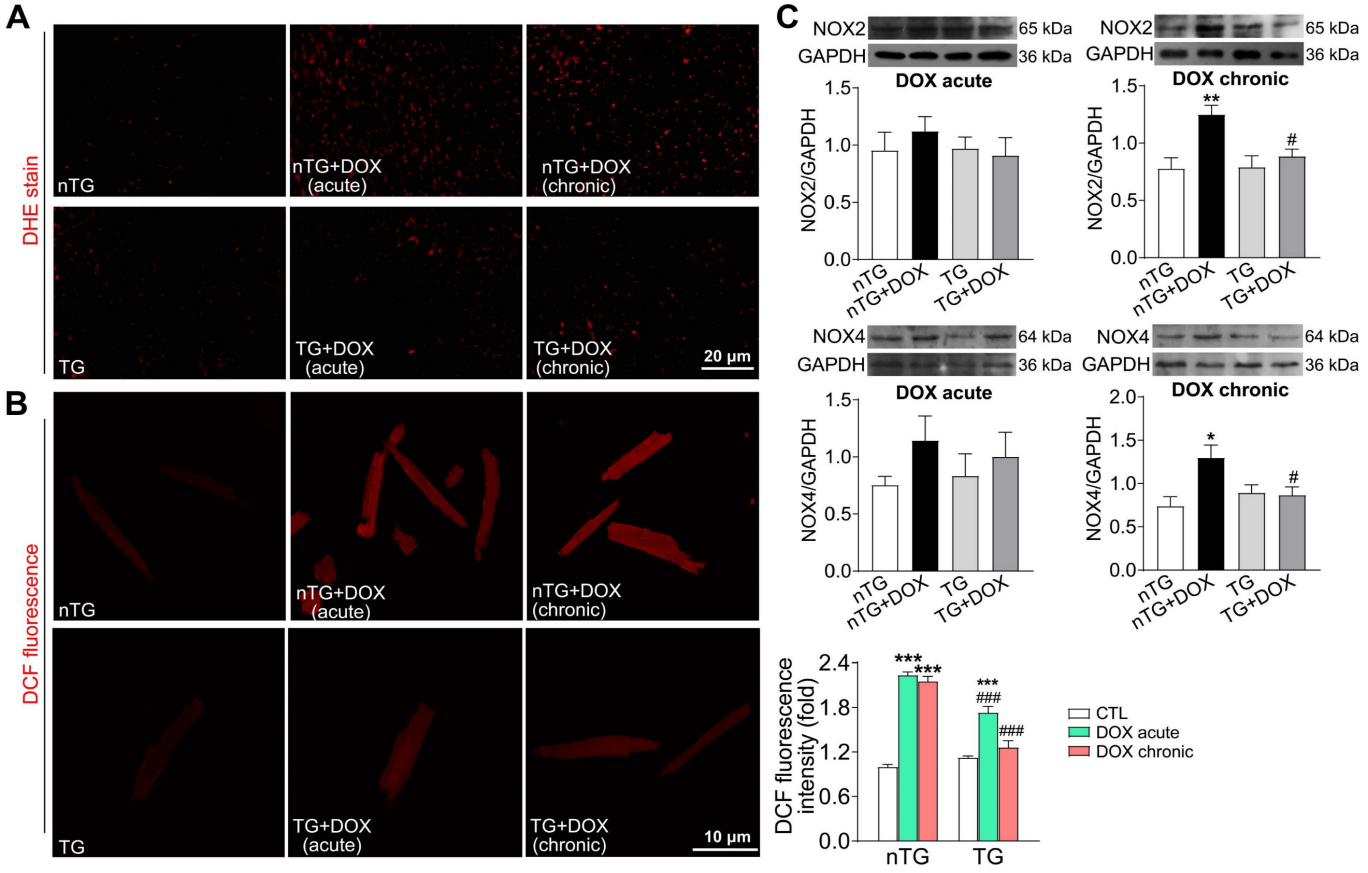
** Oxidative stress level in nTG and dnMst1-TG mice received with different DOX interventions. (A)** Images of DHE staining for O_2_^-^ in mouse hearts. **(B)** Images of DCF fluorescent staining for ROS in isolated adult mouse cardiomyocytes and quantitative analysis of fluorescence intensity. n = 10-15, ^***^P < 0.001* vs.* respective control (CTL); ^###^*P* < 0.001 *vs.* nTG counterpart. **(C)** Representative immunoblotting images and quantitative analysis for NOX2 and NOX4 protein expression level in LV tissues. n = 6, ^*^P < 0.05, ^**^P < 0.01 *vs.* nTG; ^#^*P* < 0.05 *vs.* nTG+DOX. Data are presented as Mean ± SEM.

To further study the role of Hippo pathway in DOX-induced cardiomyopathy, we determined alterations in myocardial injury, fibrosis and oxidative stress. Plasma concentrations of cTnI and Gal-3 were markedly increased in mice with acute DOX treatment, evidence for myocardial injury and pro-inflammatory sign. In dnMst1-TG mice, whilst plasma Gal-3 level was significantly high with chronic DOX, acute DOX-evoked increase in both cTnI and Gal-3 were lower relative to nTG control (Figure 7A). Masson trichrome staining revealed increased collagen deposition in hearts of mice with chronic DOX treatment, the change associated with upregulated expression of fibrotic proteins Gal-3, collagen I and connective tissue growth factor (CTGF). All these alterations were significantly mitigated in dnMst1-TG hearts versus nTG counterparts (Figure 7B-C). Furthermore, both acute and chronic DOX regimens markedly enhanced myocardial oxidative stress measured by O_2_^-^ or ROS, and key ROS generating enzymes NOX2 and NOX4. These DOX-induced changes were significantly attenuated in dnMst1-TG (Figure 8A-C).

### Chronic verteporfin treatment effectively inhibited myocardial YAP activity

**Figure 9:**
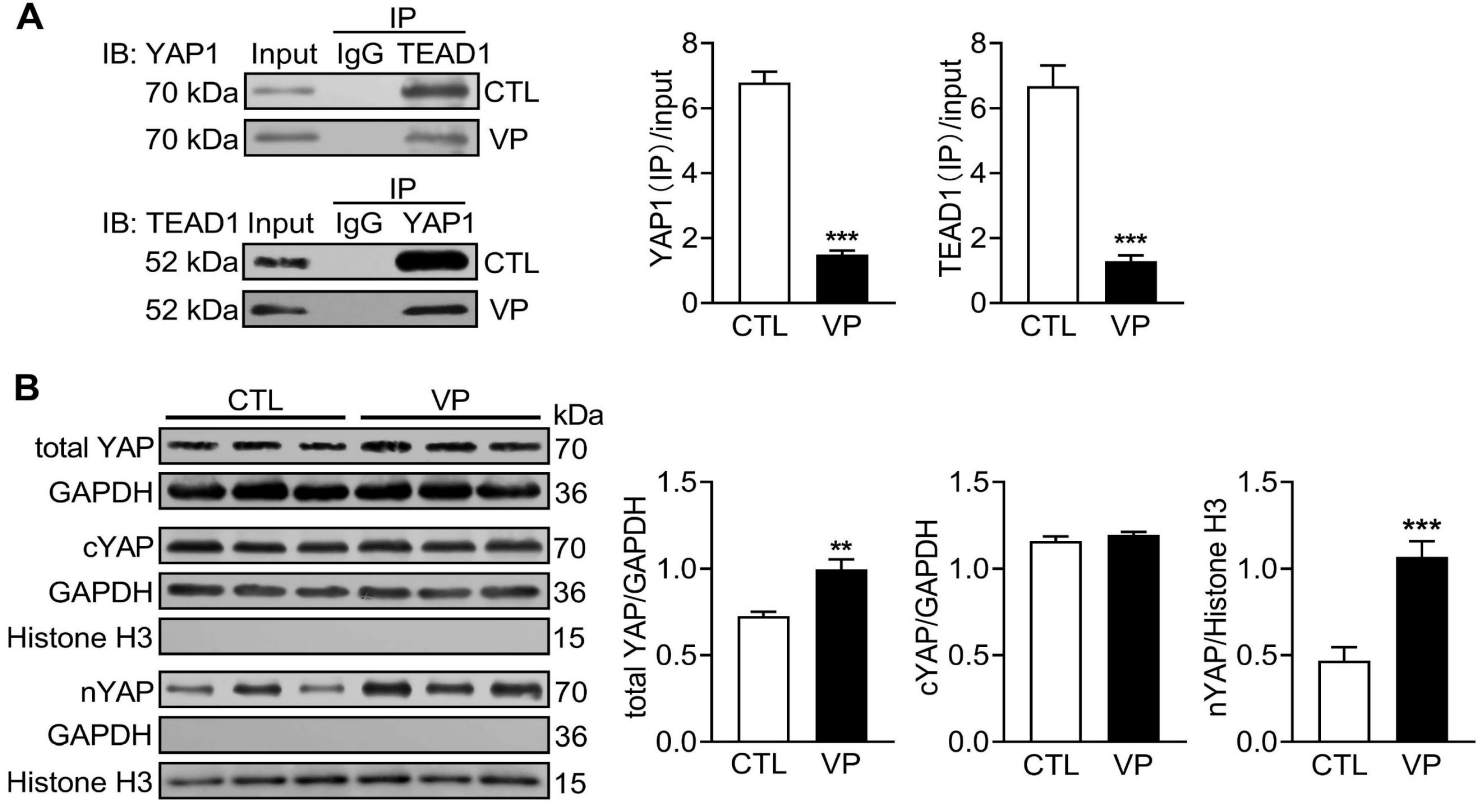
** Changes of myocardial YAP level and its nuclear interaction with TEAD1 in mice without or with treatment of YAP inhibitor verteporfin.** C57BL/6 mice were administrated with or without verteporfin (VP, 100 mg/kg, i.p. once every other day) for 2 months.** (A)** Representative immunoblots and quantitative analysis of nuclear protein co-immunoprecipitation for YAP1-TEAD1 interaction in mouse heart.** (B)** Representative immunoblotting images and quantitative analysis of total YAP, cytoplasmic YAP (cYAP) and nuclear YAP (nYAP) in mouse hearts. GAPDH was used as total and cytoplasmic protein marker, histone H3 was used as nuclear marker. Data are presented as Mean ± SEM, n = 6, ^**^P < 0.01, ^***^P < 0.001 *vs.* control (CTL).

Verteporfin is a new anti-cancer drug that suppresses nuclear YAP-TEAD interaction 16. C57/BL6 mice were treated with verteporfin for a period of 2 months and soon YAP activity were studied in myocardium. Co-immunoprecipitation of extracted LV tissue nuclear protein showed reduction in physical interact of nuclear YAP and TEAD1 in verteporfin-treated hearts which indicated inhibition of YAP-TEAD1 transcriptional activity by verteporfin (Figure 9A). Total YAP expression in LV tissue and its level in cytoplasm and nucleus were determined by immunoblotting. Total YAP expression was significantly increased (about 25%) in verteporfin-treated hearts. Using cytoplasmic and nuclear protein fractions, nuclear YAP abundance in verteporfin-treated hearts was 2-fold of that in control hearts, while cytoplasmic YAP abundance did not change (Figure 9B). These data demonstrated effective inhibition of myocardial YAP by verteporfin treatment.

### Chronic verteporfin treatment induced cardiac fibrosis, oxidative stress and dysfunction

**Figure 10:**
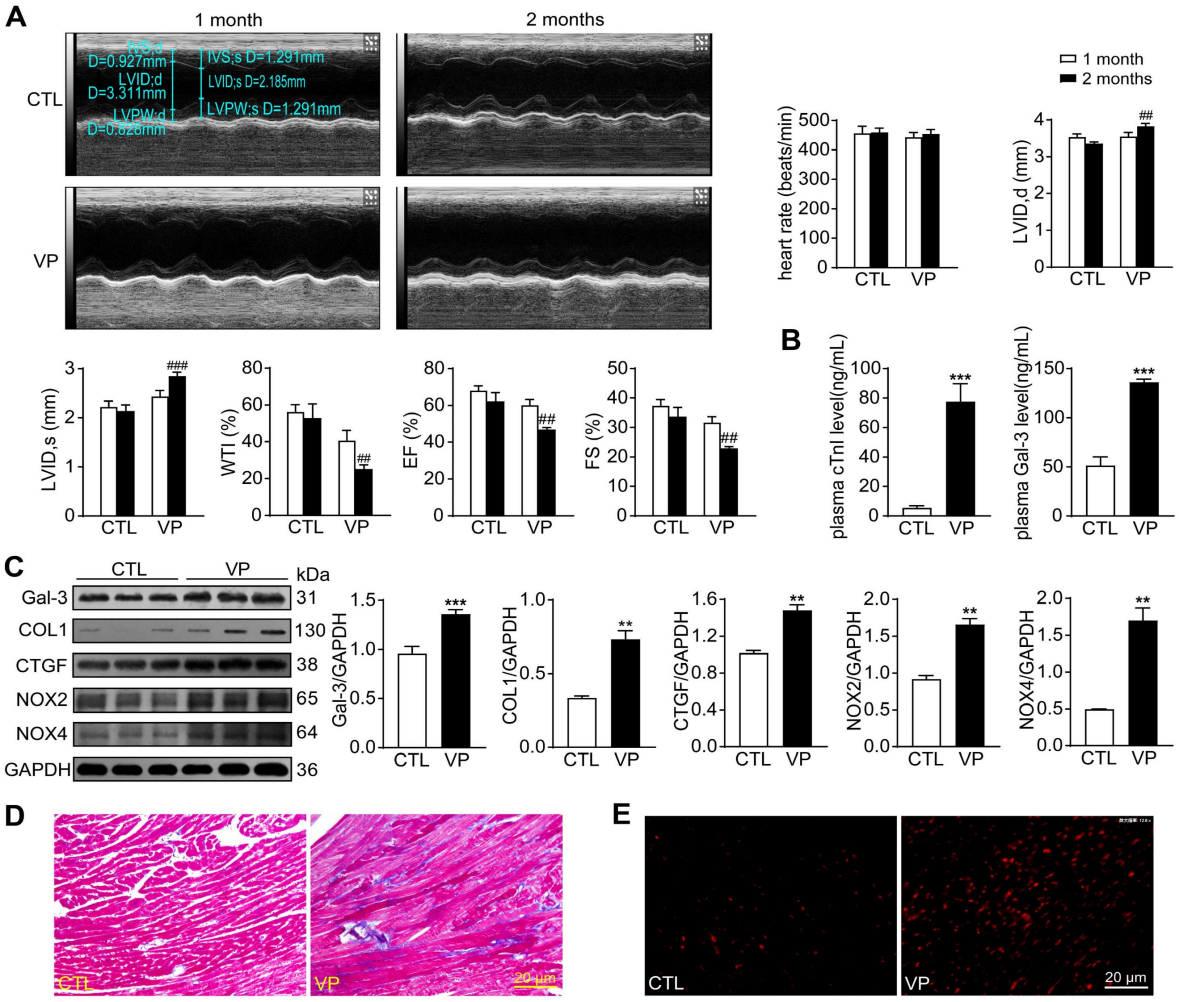
** Alterations of myocardial injury markers, left ventricular function, cardiac fibrosis and oxidative stress in mice without or with treatment of verteporfin. (A)** Representative M-mode echocardiographic tracings from short-axis LV 2-dimensional images of mice without or with VP treatment for 1 and 2 months, separately. Heart rate, LV internal diameter at end-diastole (LVID, d), LV internal diameter at end-systole (LVID, s), wall thickening index (WTI), ejection fraction (EF) and fractional shortening (FS) were derived from LV ultrasound images (n = 8-10 in each group). **(B)** Plasma levels of myocardial injury markers cTnI and galectin-3 (Gal-3). **(C)** Representative immunoblotting images and quantitative analysis for Gal-3, collagen I (COL1), CTGF, NOX2 and NOX4 protein expression level in mouse hearts. **(D)** LV sections with Masson trichrome staining for collagen (blue). **(E)** Images of DHE staining in LV sections. Data are presented as Mean ± SEM, n = 6, ^**^P < 0.01, ^***^P < 0.001 *vs.* control (CTL); ^##^*P* < 0.01, ^###^*P* < 0.001 *vs.* CTL 2 months.

Potential cardiotoxicity of verteporfin was further explored in wild-type mice. Echocardiographic tests were performed at 1- and 2-month of the study period to monitor cardiac function along the course of verteporfin treatment. Heart rate, LV dimensions and contractile parameters were stable in vehicle-treated mice. Verteporfin treatment resulted in a time-dependent onset of LV dysfunction with increase in LV internal diameters and decline in wall thickness, ejection fraction and fractional shortening only at the 2-month time point (Figure 10A). Mice received verteporfin treatment had increased plasma levels of cTnI and Gal-3 (about 8-fold and 2.5-fold increase, Figure 10B). Establishment of cardiac fibrosis by verteporfin treatment was indicated by Masson trichrome staining and elevated expression of Gal-3, collagen I and CTGF. Meanwhile, verteporfin treatment significantly increased cardiac levels of O_2_^-^ and expression of NOX2 and NOX4 (Figure 10C-E).

### Pharmacological YAP inhibition with verteporfin led to structural and functional abnormalities of cardiac mitochondria

**Figure 11:**
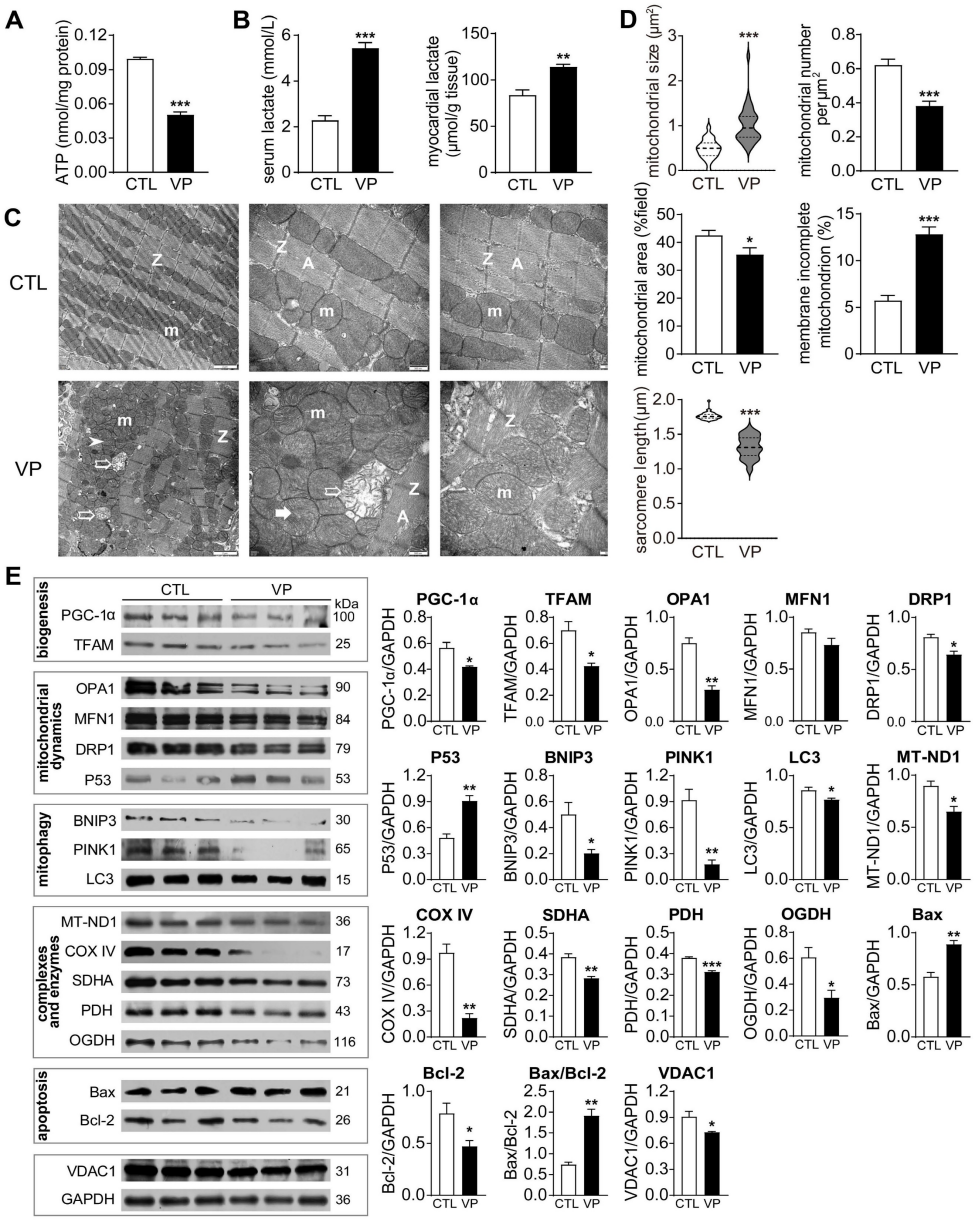
** Effect of verteporfin treatment on cardiac mitochondrial metabolism, ultrastructure and turnover markers in wild-type mice. (A)** ATP level in LV tissues. **(B)** Lactate level in serum and LV tissues.** (C)** Electron microscopic images of the LV myocardium. From left to right: magnification at 4,000, 10,000 and 10,000, respectively. m: mitochondria; A: A-band; Z: Z-line; ➤: irregularly arranged mitochondria; *: swollen mitochondria or mitochondria with disrupted or dissolved cristae; ➯: damaged mitochondria with incomplete outer-membrane.** (D)** Quantitative measures of mitochondrial size, density and distribution, the ratio of mitochondria with incomplete outer-membrane and sarcomere length of mouse myocardium (n = 4 hearts/group). **(E)** Immunoblotting of LV tissue protein for marker proteins of mitochondrial biogenesis, fusion, fission, mitophagy, respiratory complexes I-III or TCA cycle enzymes, apoptosis and transporter VDAC1. Protein expression level was quantified and expressed as ratio relative to GAPDH. Data are presented as Mean ± SEM, n = 6, ^*^P < 0.05, ^**^P < 0.01, ^***^P < 0.001 *vs.* control (CTL).

DOX-induced cardiotoxicity was characterized by mitochondrial damage and dysfunction. To compare with verteporfin cardiotoxicity, we examined mitochondrial structure and function in hearts from mice receiving verteporfin treatment. There was significant reduction in myocardial content of ATP (about 50% decrease) together with accumulation of lactate in verteporfin group, as well as elevated serum level of lactate (about 2.5-fold increase, Figure 11A-B). By EM imaging, verteporfin treated hearts displayed disordered arrangement, swelling, partial cristae dissolution, and outer-membrane discontinuation of mitochondria, and shortened sarcomere length (Figure 11C). Quantitative analysis showed doubled mitochondrial size and reduced density by 30% in verteporfin group (Figure 11D). Expression of mitochondrial marker proteins was also examined. Verteporfin treatment significantly reduced selected marker proteins for mitochondrial biogenesis (PGC-1α and TFAM), fission and fusion (Figure 11E). Meanwhile, mitochondrial proteins reflecting mitophagy, complexes and oxidative phosphorylation function were decreased in verteporfin group. Mitochondria-dependent cell apoptosis was enhanced by verteporfin treatment (Figure 11E). Thus, our results implicated verteporfin-cardiotoxicity via inducing mitochondrial structure damage and dysfunction.

## Discussion

Using human breast cancer cell and Mst1 kinase-dead mouse model, we investigated the role of Hippo pathway in mediating chemotherapy-induced cardiomyopathy. By focusing on mitochondrial damage, our study brought some new perspectives. By inducing rapid and sustained activation of Hippo pathway, chemotherapeutic drugs DOX and verteporfin achieved their anti-cancer effect in cancer cells, but also induced cardiotoxicity and cardiomyopathy. Hippo pathway activation induced significant mitochondrial damage forming a pivotal mechanism for chemotherapy-induced effects on cancer cells and adverse effect on the heart. Collectively, our results highlight a causative role of Hippo pathway in development of mitochondrial abnormality leading to both anti-cancer efficacy and cardiomyopathy by chemotherapeutic drugs.

We conducted *ex vivo* (cancer cells) and *in vivo* experiments testing the role of Hippo-YAP signaling in mediating anti-cancer efficacy as well as cardiotoxicity. Hippo-YAP pathway was targeted in both models by using siRNA-induced Mst1-knockdown in cancer cells, Mst1 kinase-dead (dnMst1-TG) mouse model 25, and verteporfin as a specific inhibitor for YAP-TEAD transcriptional activity 28. Based on the strong evidence for activated Hippo signaling in mediating mitochondrial gene downregulation and damage 21, 23, 24, we especially explored mitochondrial damage and dysfunction in both biological systems. To simulate clinical administration with DOX, acute and chronic DOX regimens were tested, and the potential cardiotoxicity of recently developed verteporfin was simultaneously investigated *in vivo*. Our results of phenotypic changes in cancer cells or mouse hearts following exposure to both drugs or genetic interventions support the notion that Hippo pathway activation/YAP inhibition constitutes the underlying mechanism leading to mitochondrial damage and ultimately anti-cancer efficacy or cardiomyopathy. We observed cardiotoxicity of verteporfin as a new class of anti-cancer drugs, which calls for caution when this drug is used for cancer therapy in the future.

We observed in MDA-MB-231 breast cancer cells that DOX-induced cell death and viability decrease are accompanied with and also indispensable to Mst1 activation/YAP inactivation, suggesting that withdrawal of transcriptional activity of YAP is a key mechanism. This novel finding adds to our current knowledge on the anti-cancer mechanisms of DOX, including interference of DNA replication and transcription, topoisomerase II inhibition and oxidative damage 29, 30. In several malignancies, Hippo pathway is restrained while YAP activity is enhanced thereby promoting cell proliferation but inhibiting cell death 31. We showed in breast cancer cells that DOX-induced cell apoptosis and mitochondrial damage were markedly attenuated by Hippo pathway inactivation through Mst1 gene silencing, but simulated by verteporfin through inhibiting YAP transcriptional activity. Whilst we showed cancer cell death by apoptosis in mechanism, other modes of cell deaths are likely to occur in our experimental conditions, such as mitochondria-dependent ferroptosis as implicated by EM images showing condensed mitochondrial outer membrane as typically seen in cells undergoing ferroptosis 32, 33. Significant structural abnormalities of mitochondria following treatment with DOX or verteporfin likely contribute to increased cancer cell death.

While mediating anti-cancer effects, Hippo pathway activation in the myocardium contributes significantly to chemotherapy-induced cardiotoxicity. Accumulating studies have shown that Hippo pathway and YAP plays an important role in both cancerogenesis and cardiac structural and functional homeostasis 9, 17. Clinical and experimental studies have demonstrated Hippo pathway activation/YAP inhibition in various types of heart disease including DCM, diabetic cardiomyopathy, arrhythmogenic cardiomyopathy, myocardial infarction, and HF 17, 34-36. Here we demonstrated in DOX-treated mice, especially under chronic DOX administration, that activated Hippo pathway causatively associated with cardiomyopathy measured by myocardial injury, oxidative stress and fibrosis. Between the two DOX regimes, acute DOX treated mice showed more severe myocardial injury, but hearts from mice with chronic DOX treatment exhibited enhanced Hippo pathway activity, oxidative stress and fibrogenesis. Importantly, using dnMst1-TG mice as a model of inactivated cardiac Hippo pathway, cardiomyopathy induced by either acute or chronic DOX regimens was mitigated. This finding is further supported by results from verteporfin treated animals exhibiting cardiomyopathy comparable to that by DOX therapy. Although previous studies on DOX-cardiotoxicity showed cardiomyocyte apoptosis 37, our observation of elevated cardiac biomarkers like troponin-I implicates other cell death modes that associated with disruption of cellular membrane, like necrosis or ferroptosis 32, 38.

Our results clearly showed that anti-cancer drugs induced significant mitochondrial damage in cancer cells *ex vivo* and cardiomyocytes *in vivo*. Mitochondria exert diverse energetic and signaling function, most notably oxidative phosphorylation (OXPHOS), central carbon metabolism or generation of intermediate metabolites, thereby regulating cell fate and function 39. Functional mitochondria are essential for cancer genesis and cell proliferation 40. Suppressed mitochondrial bioenergetics and associated signaling activity in cancer cells would attenuate cancerogenesis. Indeed, mitochondria-targeted pharmacological interventions have been proposed as a new approach for anti-cancer therapy 41. Damaged mitochondria tend to reduce OXPHOS energy generation but increase oxidative stress and cell death signaling via apoptosis or ferroptosis 32, 42, 43. By EM imaging and various biochemical assays, we showed that following exposure to DOX or verteporfin, cancer cells exhibited significant mitochondrial damage and undergoing cell death. Notably, these cellular toxic effects of DOX were mitigated by Mst1-siRNA knockdown. Thus, Hippo pathway activation induced mitochondrial damage likely to contribute importantly to cancer cell death following chemotherapy.

In the clinical setting, use of DOX for cancer therapy is limited by a dose- and duration-dependent toxicity in many organs, most notably arrhythmia, cardiomyopathy, left ventricular dysfunction and congestive HF 4, 30. The molecular mechanism of DOX-induced cardiomyopathy is controversial and mitochondrial dysfunction has been implicated as the critical factor 44, 45. Previous studies showed that DOX has a good affinity to cardiomyocyte mitochondria by which it induces oxidative stress and expression of apoptotic proteins leading to cardiomyocyte apoptosis 30, 46. Several pieces of results in the present study proved that structural and functional damage to cardiomyocyte mitochondria constitute a major cause for DOX cardiotoxicity. First, RNA-sequencing data showed that mitochondrial genes were among the most significantly down-regulated gene sets in hearts of mice subjected to acute or chronic DOX administration. Second, DOX treatment caused distinct myocardial mitochondrial damage and reduced mitochondrial density observed via EM imaging. Third, mitochondrial dysfunction by DOX is characterized by lower myocardial ATP content but enhanced glycolysis, and downregulation of mitochondrial marker proteins of biogenesis, turnover, mitophagy and OXPHOS, together with increased Bax/Bcl2 ratio. Mitochondrial TCA enzymes like PDH and OGDH up-regulation after DOX administration also exacerbate mitochondrial dysfunction 47. Increased lipid droplets in cardiomyocytes is likely due to shut-down of mitochondria ultilization of fatty acids via OXPHOS. In cancer cells and mouse hearts exposed to anti-cancer drugs, we observed similar morphological changes in mitochondria, i.e. reduction in number and increase in size together with higher proportions of damaged mitochondria. We also observed reduction in protein abundance of relevant mitochondrial markers. These changes indicate impaired biogenesis and fission/fusion, processes essential for repairmen and renewal of damaged mitochondria. Importantly, these parameters of mitochondrial damage and cardiomyopathy phenotype induced by DOX were significantly ameliorated in dnMst1-TG mice. As far as we are aware, this is the first report on the causal role of Hippo pathway activation in mediating DOX cardiotoxicity with myocardial mitochondrial damage as a pivotal mechanism.

Using cardiomyocyte-specific Mst1 transgenic overexpression (Mst1-TG) mice at 3-week and adult ages, representing early and severe stages of DCM, we recently showed that enhanced cardiac Hippo pathway induces mitochondrial damage that causatively leads to DCM 19, 21. This finding was also supported by studies using cardiomyocyte-restricted YAP- or TEAD1-knockout mice 22-24. In the current study, we established the role of Hippo pathway activation/YAP inhibition in the development of cardiomyopathy induced by DOX or verteporfin through mediating mitochondrial damage and dysfunction. There was a high degree consistency between current study on DOX-induced cardiomyopathy and Mst1-TG model 21 in changes of Hippo pathway activation, mitochondrial damage and onset of cardiomyopathy. Mechanistically, we showed in DOX treated hearts attenuated YAP transcriptional activity. This was further confirmed by our verteporfin experiments showing that chronic inhibition of YAP-TEAD interaction led to pronounced cardiac mitochondria damage and dysfunction, oxidative stress, functional deterioration and fibrosis.

Numerous studies on cancer tissues or cells have shown that verteporfin achieves its anticancer effect through disrupting YAP-TEAD1 interaction, which was accompanied by downregulated YAP protein level 16, 48. In the heart, we observed by co-IP the same effect of verteporfin on inhibiting nuclear YAP-TEAD1 interaction. Here attenuated transcriptional regulation by YAP is further indicated by upregulated target genes like Gal-3 and CTGF, which has been previously observed in the heart as YAP is known to be transcriptional activator or repressor 21, 49. Interestingly, unlike cancer tissue or cells, verteporfin induced elevated total YAP and nYAP, and the latter might suggest increased YAP nuclear entry. Cell-type dependent changes in YAP protein expression by verteporfin deserves further investigation.

Animals receiving chronic treatment with DOX or verteporfin developed cardiac fibrosis together with sustained upregulation of pro-fibrotic molecules like Gal-3 and CTGF. Whilst previous clinical and experimental studies have shown this, the current study provided mechanistic insight into the pro-fibrotic signaling. One important finding is that fibrotic markers were suppressed in dnMst1-TG mice that express only in cardiomyocytes Mst1 transgene with kinase-dead mutation 25. Thus, activation of cardiomyocyte Hippo pathway contributes to fibrogenesis, apparently via cardiomyocyte-fibroblast interactions in the mechanism. We previously showed that cardiomyocyte and other cell types (inflammatory cells, fibroblasts) could release pro-inflammatory and pro-fibrotic factors like Gal-3 or interleukin-18 to promote fibroblast activation and cardiac fibrogenesis in cardiomyocyte-restricted genetically modified mouse models 27, 50. Supportive to this notion also comes from studies on the cardiomyocyte-restricted genetic interventions that either activate Mst1 (i.e. Mst1-TG) 19, 21 or suppress YAP or TEAD1 (i.e. YAP- or TEAD1-gene deletion) 23, 24, 51, all result in dilated cardiomyopathy with significant interstitial fibrosis.

In conclusion, the present study demonstrated that through Hippo pathway activation/YAP inactivation, chemotherapy drugs DOX and verteporfin exert cell toxicity in cancer cells, but induced overt cardiotoxicity and dysfunction. Our findings of mitochondria damage and cardiomyopathy by anti-cancer drugs provide evidence for myocardial Hippo pathway as a putative therapeutic target for limiting cardiotoxicity by chemotherapy, and also hinted the direction in developing the strategy or interventions against chemotherapy-induced cardiomyopathy, such as preventive strategies through targeting cardiac Hippo pathway/YAP activity to preserve myocardial mitochondria and limiting cardiomyopathy.

## Supplementary Material

Supplementary tables.
