# Supplementary material for: Hippo pathway activation mediates chemotherapy-induced anti-cancer effect and cardiomyopathy through causing mitochondrial damage and dysfunction
Source: Theranostics. 2023 Jan 1;13(2):560–77. doi: 10.7150/thno.79227 (PMC9830444; doi:10.7150/thno.79227)
Supplement: Supplementary file 1 — Supplementary tables. [file thnov13p0560s1.pdf]

Hippo pathway activation mediates chemotherapy-induced anti-cancer effect and cardiomyopathy through causing mitochondrial damage and dysfunction

Gang She[1]; Jin-Chan Du[1]; Wei Wu[1]; Tian-Tian Pu[1]; Yu Zhang[1]; Ru-Yue Bai[1]; Yi Zhang[1]; Zheng-Da Pang[1]; Hui-Fang Wang[2]; Yu-Jie Ren[2]; Junichi Sadoshima[5]; Xiu-Ling Deng[1, 3]; Xiao-Jun Du[1, 4*]

[1]Department of Physiology and Pathophysiology, School of Basic Medical Sciences, Xi'an Jiaotong University Health Science Center, 76 West Yanta Road, Xi'an, 710061, Shaanxi, China

[2]Department of Pathology, Xi'an People's Hospital (Xi'an Fourth Hospital), Affiliated Guangren Hospital, Xi'an Jiaotong University Health Science Center, 21 Jiefang Road, Xi'an, 710005, Shaanxi, China

[3]Cardiovascular Research Centre, School of Basic Medical Sciences, Xi'an Jiaotong University Health Science Center, 76 West Yanta Road, Xi'an, 710061, Shaanxi, China

[4]Baker Heart and Diabetes Institute, 75 Commercial Road, Melbourne, Victoria 3004, Australia

[5]Rutgers New Jersey Medical School, Department of Cell Biology and Molecular Medicine, New Jersey, United States of America.

Correspondence: XJ Du, email: xiao-jun.du@baker.edu.au

SUPPLEMENTARY MATERIALS

Table S1. Source and characteristics of antibodies used for immunoblotting

Table S2. Full list of abbreviations use in figures

Table S1. Source and characteristics of antibodies used for immunoblotting

Protein	supplier	Lot number	concentration	MW (kDa)
Mst1	CST	#3682	1:1000	59
p-Mst1	CST	#49332	1:1000	59
p-Yap	CST	#4911	1:1000	69
Yap	CST	#12395	1:1000	69
PGC-1α	proteintech	66369	1:1000	100
OPA1	CST	# 80471S	1:3000	80-100
Mfn1	abcam	ab104274	1:1000	84
DRP1	CST	# 8570S	1:1000	82
p53	abcam	ab26	1:1000	53
LC3A	CST	# 4599S	1:1000	14,16
Pink1	abcam	ab23707	1:500	66
Bnip3	abcam	ab10433	1:1000	30
MT-ND1	abcam	ab181848	1:5000	36
SDHA	CST	#11998	1:3000	70
COXIV	proteintech	11242-1-AP	1:4000	17
OGDH	abcam	ab137773	1:5000	116
PDH	CST	#3205	1:3000	43
Bax	CST	#2772S	1:1000	20
Bcl-2	CST	#3498S	1:1000	26
VDAC1	proteintech	10866-1-AP	1:4000	31
TFAM	abcam	ab131607	1:2000	25
GAPDH	proteintech	10494-1-AP	1:4000	36
CTGF	proteintech	23936-1	1:500	38
Yap1	santa	sc-376830	1:500	70
Tead1	santa	sc-393976	1:500	50
Tead1	CST	#12292	1:1000	50
Histone H3	Proteintech	17168-1-AP	1:3000	15
Galectin-3	abcam	ab2785	1:1000	31
NOX2	Biosis	bs-3889R	1:500	65
NOX4	Biosis	bs-1091R	1:500	64
Collagen I	Biosis	bs-0578R	1:500	130
IgG	Beyotime	A7028		

Table S2. Full list of abbreviations used in Figures

Fig 1 Abbreviations	Full term
Mst1	mammalian sterile-20 like kinase 1
YAP	yes-associated protein
Bax	BCL2 associated X, apoptosis regulator
Bcl-2	BCL2 apoptosis regulator
GAPHD	glyceraldehyde-3-phosphate dehydrogenase
CTL	control
DOX	doxorubicin

Fig 2 Abbreviations	Full term
VP	verteporfin

Fig 3 Abbreviations	Full term
DEG	differentially expressed gene

Fig 4 Abbreviations	Full term
nTG	non-transgenic
dnMst1-TG	dominant negative Mst1 transgenic

Fig 6 Abbreviations	Full term
PGC-1α	peroxisome proliferator-activated receptor γ coactivator 1-α
TFAM	Transcription factor A, mitochondrial
Mfn1	mitofusin 1
OPA1	optic atrophy 1, mitochondrial dynamin-related protein
Drp1	dynamin-related protein 1
Bnip3	BCL2 interacting protein 3
Pink1	PTEN induced kinase 1
LC3	microtubule associated protein 1 light chain 3 alpha
MT-ND1	NADH dehydrogenase, subunit 1 (complex I)
SDHA	succinate dehydrogenase complex flavoprotein subunit A
COX IV	cytochrome c oxidase subunit 4
OGDH	oxoglutarate dehydrogenase
PDH	pyruvate dehydrogenase
Bcl-2	BCL2 apoptosis regulator
Bax	BCL2 associated X, apoptosis regulator
VDAC1	voltage-dependent anion channel 1
GAPDH	glyceraldehyde-3-phosphate dehydrogenase

Fig 7 Abbreviations	Full term
cTnI	Cardiac troponin I
Gal-3	Galectin-3
COL1	Collagen I
CTGF	Connective tissue growth factor

Fig 8 Abbreviations	Full term
NOX2	NADPH oxidase 2

NOX4	NADPH oxidase 4

**Fig 9 Abbreviations**	**Full term**
TEAD	TEA/ATTS domain
IP	Immunoprecipitation
IB	Immunoblot

**Fig 10 Abbreviations**	**Full term**
WTI	Wall thickening index
LVID	Left ventricular internal diameter
EF	Ejection fraction
FS	Fractional shortening
